# Entrepreneurial experimentation in business model dynamics: Current understanding and future opportunities

**DOI:** 10.1007/s11365-023-00836-7

**Published:** 2023-02-22

**Authors:** Silvia Sanasi

**Affiliations:** Centre for Family Business Management, Faculty of Economics and Management, Free University of Bozen-Bolzano, Universitätsplatz 1, 39100 Bozen-Bolzano, Italy

**Keywords:** Entrepreneurial experimentation, Business model dynamics, Business model innovation, Validation, Scaling, Pivots, Lean startup

## Abstract

The concept of business model dynamics has been gaining momentum in the academic literature to refer to all the alterations in a firm’s business model. This study taps into the shift from the traditional ontological view of business models as the static implementation of a firm’s strategy, toward a phenomenological stance that portrays the business model as a unit of analysis for different phenomena related to it. Building on this emerging discourse, this review offers an interpretive lens on the role of entrepreneurial experimentation in business model dynamics, namely business model innovation, validation, scaling, and pivots. This study proposes a unified framework for understanding these phenomena, discusses the research gaps emerging from this perspective, and advances a set of open research avenues to inform future research. The study also taps into the recent managerial interest in methods involving experimentation, such as the Lean Startup method.

## Introduction

The business model is an established construct in strategic management and entrepreneurship (Demil et al., 2015), seen as the unit of analysis to describe the realization of a firm’s strategy (Casadesus-Masanell & Ricart, 2010; Cortimiglia et al., 2016; Zott et al., 2011) in terms of the value created for target customers, the way it is delivered to them, and the mechanisms through which the firm captures value back from the market (Teece, 2010). However, the debate regarding the theoretical definition of the business model concept is still ongoing (Massa et al., 2017; Schneckenberg et al., 2022; Wirtz et al., 2016), yet rising critiques on the legitimacy of the business model as a concept per se rather than just representing “strategy in new bottles” (e.g., Bigelow & Barney, 2021; Massa et al., 2017).

On the other hand, an increasing body of research recognizes the business model as a potential source of innovation in and of itself (e.g., Casadesus-Masanell & Zhu, 2013; Spieth et al., 2014; Teece, 2010), encouraging further theorization and investigation of the business model innovation phenomenon (Foss & Saebi, 2017). Recent studies suggest that the view of the business model as a static picture of the logic of a firm may be the source of the doubts arising in the current literature; conversely, they advance a dynamic view of the business model as a tool to address change and development processes taking place within the firm (Achtenhagen et al., 2013; Demil & Lecocq, 2010). The investigation of so-called *business model dynamics* (Foss & Saebi, 2018) overcomes the ontological perspective adopted by the current debate on the business model which, up to now, failed to grant full legitimacy to the business model’s existence as a concept of its own. Rather, by adopting a phenomenological stance, the study of business model dynamics elevates the business model to the unit of analysis for evolutionary phenomena related to a firm’s strategy (Demil & Lecocq, 2010; Foss & Saebi, 2018). Within business model dynamics, the business model works as a device for managers and entrepreneurs “to explore a market and to bring their innovation–a new product, a new venture and the network that supports it–into existence” (Doganova & Eyquem-Renault, 2009, p. 1560).

According to the extant literature, business model dynamics refer to all the alterations to the firm’s business model that enable it to produce sustained value creation throughout time (Achtenhagen et al., 2013; Foss & Saebi, 2018), such as the developmental or change processes taking place in both entrepreneurial and incumbent firms (Schneckenberg et al., 2022). Business model dynamics may for example encompass *business model innovation*, aimed at discovering new value creation and capture opportunities (e.g., Andries et al., 2013; Foss & Saebi, 2018; Zott et al., 2011), *business model validation,* accomplished to ensure the viability of a firm’s business model choices (e.g., Eisenmann et al., 2012; Gans et al., 2019; McDonald & Eisenhardt, 2020; Shepherd & Gruber, 2021; Silva et al., 2021), *business model scaling* efforts, to grow the business model following its market validation (e.g., Nielsen & Lund, 2018; Picken, 2017), as well as the *pivots* firms set in place in their business model to face adverse events (e.g., Berends et al., 2021; McDonald & Gao, 2019; Pillai et al., 2020; Kirtley & O'Mahony, 2023).

Yet, the literature on business model dynamics is still scant and lacks a unified framework that can support theory building. In fact, the majority of the existing literature has adopted an ontological perspective in the investigation of business models (e.g., Massa et al., 2017), that has long “trapped” researchers into the debate over terminological and definitional issues on the business model construct and prevented building cumulative knowledge (Foss & Saebi, 2018). Looking at business model dynamics through a phenomenological lens, on the other hand, may give rise to novel ways of employing the business model as a unit of analysis for analyzing multiple strategy-related phenomena involving firms. This perspective may, in turn, stimulate the reflection on how business model dynamics take place within firms. In this sense, a growing body of scholarly and managerial accounts points towards experimentation with the firm’s business model as the way through which business model dynamics are enacted (e.g., Andries et al., 2013; Berends et al., 2016; Bojovic et al., 2018; Camuffo et al., 2020; Chesbrough, 2010; Ghezzi & Cavallo, 2020; McDonald & Eisenhardt, 2020; McGrath, 2010). These accounts build on established concepts in the management literature (e.g., McGrath, 1999; Murray & Tripsas, 2004; Sarasvathy, 2001) to bridge the knowledge gaps related to the phenomena involving a firm’s business model.

Experimentation originated from the entrepreneurship world (Gans et al., 2019) as a response to the high levels of uncertainty new ventures encounter (Loch et al., 2008; Rindova & Courtney, 2020; Zellweger & Zenger, 2021) and the need to “make do” with the limited resources at their disposal (Baker & Nelson, 2005; Katila & Shane, 2005). Yet, experimentation is not only a new venture’s matter. Established firms may also find themselves dealing with ever-evolving environments and unforeseeable conditions (Brown & Eisenhardt, 1997; Lichtenstein et al., 2007; Sosna et al., 2010), such as when launching novel business models (Doz & Kosonen, 2010; McGrath, 2010), calling for rapid and continuous experimentation (Berends et al., 2016; Chesbrough, 2010; Chesbrough & Tucci, 2020; Hampel et al., 2020b).

Experimentation involves the application of a rigorous and almost “scientific” method (Camuffo et al., 2020; Murray & Tripsas, 2004; Silva et al., 2021) to validate the key assumptions underlying the firm’s business model (Bocken & Snihur, 2020; Frederiksen & Brem, 2017; Gambardella & McGahan, 2010; Ghezzi, 2020; Shepherd & Gruber, 2021). Similarly to natural scientists, who employ the scientific method to test (and potentially falsify) their theories about nature (Popper, 1963), managers and entrepreneurs become theorists in search of validation of the hypotheses they formulated regarding their business and its potential viability on the market (Agrawal et al*.*, 2021; Felin & Zenger, 2009, 2017; Zellweger & Zenger, 2021).

In practice, experimentation in business model dynamics is translated into running experiments on business model alternatives (Andries et al., 2013; Gans et al., 2019), embodied into business model hypotheses (Shepherd & Gruber, 2021), before committing significant resources to any (Chesbrough, 2010; McGrath, 2010). After evaluating the results of their experiments, firms must decide whether their hypotheses are falsified and pivot their business model, revising some of its key elements to match the newly found knowledge on its underlying assumptions (Bocken & Snihur, 2020; Frederiksen & Brem, 2017; Gambardella & McGahan, 2010; Leatherbee & Katila, 2020) or, in case they are validated, to continue and persevere with the business model as planned (Eisenmann et al., 2012).

However, the current body of literature lacks the necessary “cumulativeness” for theory building (Foss & Saebi, 2018; Silva et al., 2020) on the use of experimentation to enact different business model dynamics, and existing studies are disproportionately focused on selected business model dynamics (i.e., business model innovation) (e.g., Berends et al., 2016; Chesbrough, 2010). As a consequence, the scholarly understanding of experimentation across business model dynamics appears fragmented and obstacles theory building. To bridge this gap, this study aims to provide a unified framework that can support the current scholarly understanding of the way firms can enact experimentation across business model dynamics. In particular, our study sets the objective of building the necessary cumulativeness to facilitate theory building, recognizing patterns in the existing body of knowledge, spotting relevant gaps in the literature, and advancing potential avenues for future investigation.

This study’s contribution is twofold. On the one hand, it provides an original contribution to the literature on business models (e.g., Foss & Saebi, 2017; Massa et al., 2017) by drawing a taxonomy of phenomena and systematizing them within an interpretive framework that may prospectively facilitate theory building. On the other hand, this study ignites and reunites the emerging literature on business model dynamics with the most recent debate on entrepreneurial experimentation (e.g., Agrawal et al*.*, 2021; Zellweger & Zenger, 2021), which had started growing apart. This way, it sets the ground for future research avenues that leverage on this awareness by highlighting the gaps that emerge from this overlap.

The following sections are organized as follows. First, the method section illustrates the methodological stance adopted in this review and the selection criteria for the articles included in this study. The next section provides a brief overview of the state-of-the-art research on business model dynamics. Within this section, informed by extant research, the article presents four business model dynamics. In particular, this study critically reflects on business model innovation, validation, scaling, and pivots, and the role of entrepreneurial experimentation within each of them. Building on these four dynamics, the following sections present the most prominent gaps within the current scholarly debate and some illustrative research questions that stem from each of them. The article ends with some concluding remarks, this study’s implications for scholarship and practice, as well as some emerging limitations.

## Method

﻿This article presents a narrative literature review to examine the landscape on entrepreneurial experimentation in business model dynamics. I selected this methodology with the purpose of providing a unified framework on the phenomenon and build theoretical cumulativeness by unveiling emerging patterns and literature gaps, synthesizing scholarly understanding from different communities of practice that employed different terminologies to refer to the phenomenon under scrutiny (Cronin & George, 2020; Post et al*.*, 2020; Snyder, 2019). The review is based on academic articles published between 1995 and 2023 in leading management journals covering the fields of entrepreneurship and strategic management (e.g., Academy of Management Journal, Administrative Science Quarterly, Strategic Management Journal, Strategic Entrepreneurship Journal, Academy of Management Annals, Journal of Management Studies, Entrepreneurship Theory and Practice, Strategic Organization, Long Range Planning).

Consistently with previous reviews (e.g., Cavallo et al., 2019; Schneckenberg et al., 2022) the selection of articles to be included in the review was conducted using a semi-systematic approach and followed multiple steps. The first step for the identification of the articles was an exploratory search in the Scopus online database, searching for scientific articles on “business models” that also included the term “experimentation” in their title, abstract or keywords, to refer to articles referring to experimentation as an entrepreneurial approach (as opposed to experimentation as a research method) (e.g., Andries et al., 2013). However, multiple contributions in the resulting pool of documents were building on articles that employed a slightly different terminology to refer to the use of experimentation as an approach to entrepreneurship and/or strategic management in general (e.g., Camuffo et al., 2020; Murray & Tripsas, 2004). Consequently, as a second step, I expanded the scope of the search through a snowballing approach, leveraging on the articles’ references. To ensure full coverage of the pertinent and relevant literature, I also surveyed other articles citing particularly relevant articles in this domain and evaluated their inclusion case by case. At every step of the search, I limited the selection to empirical and review articles published in academic journals included in the Academic Journal Guide of the Chartered Association of Business Schools,^1^ as it constitutes a widely accepted proxy for scientific output quality within the management community. Finally, once I had built and examined a significant body of articles on entrepreneurial experimentation in business model dynamics, I further expanded the scope of the search to adjacent concepts and theories within the reference domains of strategic management and entrepreneurship (e.g., Bortolini et al., 2018). This way, the review builds on core ideas and concepts from these literatures to shed light on important gaps and inconsistencies in the existing understanding of entrepreneurial experimentation in business model dynamics.

The review proposes a critical perspective on the articles that directly or tangentially pertain to this area of research, in the attempt to build cumulativeness and provide a unifying framework of the current understanding of entrepreneurial experimentation across different business model dynamics. In particular, most efforts were devoted to recognizing emerging patterns within the existing scholarly debate, which served to spot relevant gaps for the purpose of proposing a research agenda that can inform future studies in this domain (Breslin & Gatrell, 2023).

**Fig. 1:**
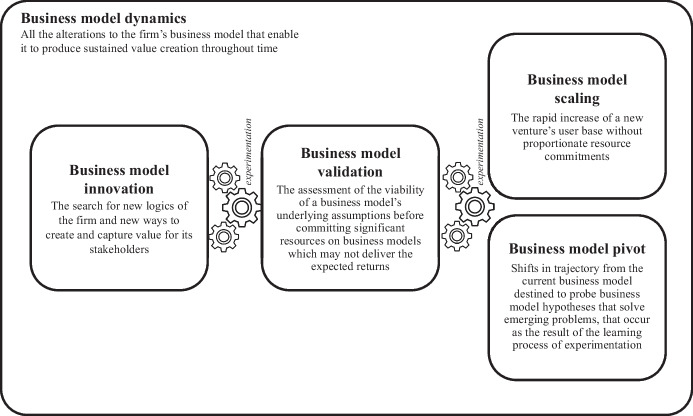
A unified framework of experimentation in business model dynamics

The following sections present the result of this review. First, the article surveys the overall literature on business model dynamics and the individual dynamics emerging from the current scholarly debate and proposing an interpretive framework (Fig. 1). Second, the emerging gaps stemming from this framework are presented and used to formulate a set of research questions to guide future research efforts.

## Business model dynamics

According to the extant body of literature, business model dynamics refer to “how business models come into being (…) and the changes in the architecture between business model elements that produce alterations to the business model” (Foss & Saebi, 2018, p. 17), as well as “shaping, adapting and renewing the underlying business model of the company” for sustained value creation (Achtenhagen et al., 2013, p. 427). The business model, thus, is no longer seen as a description of the logic of the firm in a static manner: rather, it constitutes a device that can describe and shape the development and change processes taking place within both established firms and new ventures (Doganova & Eyquem-Renault, 2009) by looking across time (Schneckenberg et al., 2022), leading their conceptualization from a phenomenological perspective. As business models are constantly subjected to re-evaluation for the firm to navigate through a changing environment to produce sustained competitive advantage (Teece, 2010), business model dynamics encapsulate the prospective character of the business model concept, highlighting its role as a market device that enable firms to evaluate and validate the future value creation and capture potential it will entail (Doganova & Eyquem-Renault, 2009).

In this sense, business model dynamics endow the business model with a performative function (Doganova & Eyquem-Renault, 2009): the business model becomes the medium that embodies business opportunities and conveys them to target stakeholders, acting as an interface between the firm and the stakeholders surrounding it (Berglund et al., 2020). At the start of any business endeavor, be it for new opportunity identification or strategic renewal, firms know little and assume much, operating in high contextual uncertainty where information is not only unknown (Loch et al., 2008), but often yet to be created (Chesbrough, 2004; Rindova & Courtney, 2020). To fight such issue, firms need to efficiently collect information creating the knowledge they miss, for example anticipating foresighted market feedback (McGrath & MacMillan, 1995). In this context, the business model thus serves the purpose to frame a new business opportunity as a set of operational hypotheses which then need to be subjected to validation, modification, or even rejection (Sull, 2004).

A growing number of studies are addressing the way firms carry out business model dynamics within the context of opportunity identification and strategic renewal (e.g., Berends et al., 2016; Bojovic et al., 2018; Ghezzi & Cavallo, 2020). In particular, they refer to the way firms enact business model dynamics as experimentation (e.g., Andries et al., 2013; Chesbrough, 2010; Garud & Karunakaran, 2018; McGrath, 2010; Murray & Tripsas, 2004). Experimentation involves a scientific attitude (Camuffo et al., 2020) toward business model dynamics: entrepreneurs and managers act like scientists, formulating hypotheses related to their business model's underlying assumptions (Eisenmann et al., 2012; Frederiksen & Brem, 2017; Gambardella & McGahan, 2010; Shepherd & Gruber, 2021), which then need to be tested against market feedback (McDonald & Eisenhardt, 2020; Thomke, 2020) in sought of validation (Frederiksen & Brem, 2017; Ghezzi & Cavallo, 2020). As a result of testing, managers and entrepreneurs are able to assess whether their business model hypotheses were validated, and decide to persevere with their initial plan (Berends et al., 2021; Leatherbee & Katila, 2020), or falsified, which may then require them to revise their business model’s core assumptions through a pivot (Kirtley & O'Mahony, 2023; Pillai et al., 2020), or abandon the endeavor entirely (Camuffo et al., 2020). The result of this testing process constitutes the so-called “validated learning” (Shepherd & Gruber, 2021).

Experimentation as a means to enact business model dynamics originated from the entrepreneurship world (Gans et al., 2019) as a response to high levels of uncertainty (Loch et al., 2008; Zellweger & Zenger, 2021) and the need to “make do” with the limited resources at the firm’s disposal (Baker & Nelson, 2005; Katila & Shane, 2005). However, experimentation is not only a new venture’s matter. Established firms may also find themselves dealing with ever-evolving environments and unforeseeable conditions while navigating through different business model dynamics (Brown & Eisenhardt, 1997; Lichtestein et al*.*, 2007; Sosna et al., 2010), such as when launching novel business models (Doz & Kosonen, 2010; McGrath, 2010), calling for rapid and continuous experimentation (Berends et al., 2016; Chesbrough, 2010; Chesbrough & Tucci, 2020; Hampel et al., 2020b).

The following sections aim at covering state-of-the-art research on different business model dynamics, delving deeper into the current understanding of each and their link to experimentation while raising the most urgent research gaps. Table 1 also provides a visual overview of the business model dynamics covered, a short definition, and its key references.

**Table 1:** Summary of the theoretical concepts identified in the literature on experimentation in business model dynamics

Concept	Definition	Main references
*Business Model Dynamics*	All the alterations to the firm’s business model that enable it to produce sustained value creation throughout time	Achtenhagen et al., 2013; Demil & Lecocq, 2010; Doganova & Eyquem-Renault, 2009; Foss & Saebi, 2018
*Business Model Innovation*	The search for new logics of the firm and new ways to create and capture value for its stakeholders	Andries et al., 2013; Berends et al., 2016; Casadesus-Masanell & Zhu, 2013; Chesbrough, 2010; Chesbrough & Tucci, 2020; Foss & Saebi, 2017; Hampel et al., 2020b; Massa et al., 2017; McGrath, 2010; Sanasi et al*.*, 2022; Schneckenberg et al., 2022; Teece, 2010
*Business Model Validation*	The assessment of the viability of a business model’s underlying assumptions before committing significant resources on business models which may not deliver the expected returns	Bocken & Snihur, 2020; Chesbrough, 2010; Gambardella & McGahan, 2010; Gans et al., 2019; Ghezzi & Cavallo, 2020; McDonald & Eisenhardt, 2020; Shepherd & Gruber, 2021; Silva et al., 2020, 2021
*Business Model Scaling*	The rapid increase of a new venture’s user base without proportionate resource commitments	Blank, 2013; DeSantola & Gulati, 2017; Eisenmann, 2021a, b; Eisenmann et al., 2012; Eisenmann & Wagonfeld, 2012; Huang et al., 2017; Nielsen & Lund, 2018; Picken, 2017; Shepherd and Patzelt, 2022; Teece, 2018
*Business Model Pivots*	Shifts in trajectory from the current business model destined to probe business model hypotheses that solve emerging problems, that occur as the result of the learning process of experimentation	Berends et al., 2021; Contigiani & Levinthal, 2019; Ehrig and Foss, 2022; Flechas Chaparro & de Vasconcelos Gomes, 2021; Frederiksen & Brem, 2017; Hampel et al., 2020a; Grimes, 2018; Kirtley & O'Mahony, 2023; McDonald & Gao, 2019; Newman et al., 2022; Pillai et al., 2020; Sala et al., 2021; Sanasi & Ghezzi, 2022; Snihur & Clarysse, 2022

First, this review considers the *innovation* of the firm’s business model, intended as the process of finding “new logics of the firm” (Casadesus-Masanell & Zhu, 2013, p. 464) aimed at discovering new value creation and capture opportunities (e.g., Foss & Saebi, 2018; Zott et al., 2011) through the novel modification of specific elements of the business model and the architecture linking them (Foss & Saebi, 2017). business model innovation, although widely studied, has not yet gained full credibility in the management literature. This shortcoming has been addressed underlining one of the greatest gaps present in the business model innovation literature, which is the lack of clear-set boundary conditions for its validity (Foss & Saebi, 2017).

Second, the study examines what previous studies reported as business model *validation.* Particularly studied in the context of nascent ventures (e.g., Eisenmann et al., 2012; McDonald & Eisenhardt, 2020), business model validation encompasses the actions undertaken by firms to evaluate the viability of their business model choices (e.g., Eisenmann et al., 2012; Gambardella & McGahan, 2010; Ghezzi, 2019; Silva et al., 2021). New ventures validate their business model looking for evidence to verify or falsify their business model’s underlying hypotheses leveraging market feedback and testing (Camuffo et al., 2020; Sull, 2004). However, although business model validation has been investigated from an individual (e.g., Grimes, 2018) and process (e.g., McDonald & Eisenhardt, 2020; Silva et al., 2021) perspective, current literature is still lagging on the investigation of how new ventures structure experimentation to achieve business model validation from an organizational perspective.

Once assessed its market validation, the business model may be ready to be grown to a wider audience, for example expanding the customer segments it is targeting, following a process of *scaling*. In this regard, I then address the rapidly growing body of literature devoted to business model *scaling*, intended as the efforts deployed by companies, mostly new ventures, to grow their business model following its validation (e.g., Nielsen & Lund, 2018; Picken, 2017). As this is one of the most threatening and crucial moments, especially for new ventures, in a business model’s lifecycle (Eisenmann, 2021a, b), business model scaling should be devoted careful attention; however, business model scaling still lacks proper investigation and, while several scholars call for its theorization, it still lacks clear definition and positioning within the strategy and entrepreneurship literature.

Finally, this review looks into the growing body of studies–particularly triggered by the emergence of the COVID-19 crisis addressing the dynamics of *pivoting* a firm's business model when faced with the failure of its underlying logic, either because of missed market validation or because its fundamental assumptions have fallen short due to changing environmental conditions (e.g., Berends et al., 2021; McDonald & Gao, 2019; Pillai et al., 2020; Kirtley & O'Mahony, 2023).

### Business model innovation

Among business model dynamics, the existing body of literature has mostly focused its attention on *business model innovation* (Foss & Saebi, 2017; Schneckenberg et al., 2022). business model innovation is a firm-specific phenomenon that has been defined as “the search for new logics of the firm and new ways to create and capture value for its stakeholders; it focuses primarily on finding new ways to generate revenues and define value propositions for customers, suppliers, and partners” (Casadesus-Masanell & Zhu, 2013, p. 464) and again the “designed, novel, nontrivial changes to the key elements of a firm’s business model and/or the architecture linking these elements” (Foss & Saebi, 2017, p. 201). Business model innovation can be embodied, among others, by the introduction of new value propositions, new customer segments, or revenue models. In late 2019, the Walt Disney Company, for example, innovated its business model by launching the proprietary “Disney + ” subscription online streaming platform for the fruition of content produced by the company (Sanasi et al., 2022).

The emerging literature on business model innovation in the early 2000s often conceptualized it as a means for profiting from novel technological advancements (Chesbrough & Rosenbloom, 2002), as well as finding new value creation and capture mechanisms related to the advent of digital technologies and the dot-com bubble (e.g., Magretta, 2002; Timmers, 1998). However, as the understanding of business model innovation evolved, it began influencing several related streams of literature where it served to embody a fruitful lens to investigate boundary-spanning phenomena (Schneckenberg et al., 2022). Among others, business model innovation was employed as an interpretive lens in a series of contexts, such as in the comparison between the traditional good-dominant logic of marketing versus the emerging service-dominant logic (e.g., Oliva & Kallenberg, 2003; Vargo & Lusch, 2004), or to compare and relate service and product innovation (e.g., Visnjic et al., 2016), to look at demand-based value creation as opposed to supply-based value creation (e.g., Priem et al., 2018), to investigate the recently emerging socio-economic phenomenon of the sharing economy (e.g. Sanasi et al., 2020), to analyze the determinants of customer brand perceptions (e.g., Spieth et al., 2019), as well as to investigate the value creation and capture mechanisms involved in the servitization of manufacturing firms (e.g., Sjödin et al., 2020).

However, diving into the process of business model innovation is not free of risk, often requiring the deployment of resources whose future return is far from being predictable in the short term. Indeed, business model innovation is rarely successful right as it is designed (Chesbrough, 2010; Teece, 2018), especially when firms deploy it to deal with novelty and consequent turbulence in the environment surrounding them (Doz & Kosonen, 2010; Foss & Saebi, 2017). Rather, it is the product of extensive processes of discovery and development through experimentation and evolutionary learning (Chesbrough, 2010; McGrath, 2010). Achieving business model innovation through experimentation entails the identification and challenge of assumptions underlying the novelties introduced in the business model (Doz & Kosonen, 2010; Gambardella & McGahan, 2010; Ghezzi & Cavallo, 2020) which should then be tested through the early involvement of customers and market actors (Garud & Karunakaran, 2018; McGrath, 2010), leading to potentially opposed outcomes–i.e., persevering with the envisioned business model innovation or revising it (Berends et al., 2021). The inherently experimental nature of business model innovation leads it to assume two potentially different roles: either it is viewed as an outcome (i.e., *the innovation* of the business model) or as an organizational process (i.e., *the process* of business model innovation) (Foss & Saebi, 2017). This dual view underlines how fundamental the practice of experimentation is to the notion of business model innovation, and how intertwined the two are in practice.

However, although experimentation is a fundamental antecedent to business model innovation, its enactment is not to be taken for granted. As reported by Chesbrough (2010), firms, particularly established ones (Snihur and Wiklund, 2019), encounter relevant barriers to experimentation which could significantly hinder their ability to introduce business model innovation. Building on this issue, an emerging body of studies has started hinting at the challenges of implementing business model innovation in established firms (Berends et al., 2016; Chesbrough & Tucci, 2020; Hampel et al., 2020b). For example, as one of the characteristics of any good strategy should ensure the preservation of firm reputation (Lanzolla & Markides, 2021; Porter, 1996), multiple studies have argued that barriers such as threats to a firm’s reputation may hinder the use of experimentation for introducing any business model innovation (Contigiani & Levinthal, 2019; Gans et al., 2019; Ghezzi, 2019). Recent studies (Sanasi et al., 2022) have addressed the issue, underlining how reputational threats may affect the way firms carry out experimentation business model innovation by building on the concept of entrepreneurial copycats (Frankenberger & Stam, 2020) to de-risk the assumptions connected to business model innovation.

Previous accounts have often encountered the issue of measuring business model innovation, in terms of radicalness of the changes performed following experimentation. Camuffo et al. (2020) study proposes to assess how radical an innovation introduced to a venture’s business model has been, according to whether it pertains the core value proposition of the product and service offered, or the target customer segments served by the venture. Similarly, Sanasi et al. (2022) found that firms may limit experimentation to less risky elements of their business model when introducing a business model innovation. This argument resonates with Ries’ (2011) idea of risky assumptions, encouraging entrepreneurs to test their business model’s riskiest assumptions first. However, the current scholarly understanding is still scant in identifying different degrees of business model innovation and how such–and other–differences may drive some boundary conditions for the use of entrepreneurial experimentation when conducting business model innovation. As such, to make up for the current lack of cumulativeness and proper theorizing in research about business model innovation, I build on the gap identified by Foss and Saebi (2017) to contend that developing a clearer understanding of the boundary conditions of the phenomenon, particularly in contexts where significant barriers to experimenting to achieve business model innovation are present, could support and facilitate theory building (Busse et al., 2017).

### Business model validation

Firms survive by constantly adapting themselves (Brown & Eisenhardt, 1997)–i.e., their business models (Teece, 2010)–and introducing discontinuities in the market (Garud & Karunakaran, 2018). This is particularly true for new ventures: in the early stages of their development, they frequently undergo frequent and severe changes in their content and structure (DeSantola & Gulati, 2017; Ghezzi & Cavallo, 2020). However, business models are rarely right at the first attempt (Teece, 2010). For this reason, as new ventures operate in highly complex environments (Nambisan, 2017) and under severe resource constraints (Busch & Barkema, 2022; Katila & Shane, 2005), they need to assess the viability of the underlying assumptions of their business model (Camuffo et al., 2020; Gambardella & McGahan, 2010; Kerr et al., 2014; Sull, 2004) to ensure fit with the market (Eisenmann et al., 2012; Shepherd & Gruber, 2021) before committing significant resources to business models which may not deliver the expected returns (Gans et al., 2019; McGrath, 1999). Dropbox, for example, experimented with its value proposition–i.e., instantaneous file sharing through a computer’s operating system’s default file manager–by measuring waitlist subscriptions following an illustrative video of what the service would look like once implemented before moving on to its development (Ries, 2011).

This process has been referred to by previous studies as business model *validation* (Eisenmann et al., 2012; Felin et al., 2019; Ghezzi & Cavallo, 2020; Silva et al., 2021). Business model validation closely relates to the principles of hypothesis validation (or falsification) as an outcome of experimentation (Bocken & Snihur, 2020; Shepherd & Gruber, 2021; Silva et al., 2020; Sull, 2004) that characterize the historical scientific method (e.g., Popper, 1963). More generally, any business opportunity can be translated into a business model, which underlies a hypothesis that may be subject to validation (Sull, 2004). In particular, new ventures validate their business model looking for evidence to verify or falsify their business model’s underlying hypotheses leveraging on experiments that rely on the collection of market feedback and testing of multiple metrics related to the business model’s success (Camuffo et al., 2020; Contigiani & Levinthal, 2019; Sull, 2004). When evaluating the outcome of the experiments conducted, previous studies reported that firms may deem their business model as viable if the results of their experiments prove its potential to earn positive revenues, or its capability to grant the acquisition and/or activation of new customers (Camuffo et al., 2020). Firms are said do so to assess their business model’s product-market fit (Eisenmann et al., 2012; Ghezzi & Cavallo, 2020), an ideal awareness of whether their value proposition matches the expectations of the customer segment(s) it targets. Once the results from experimentation have been gathered and examined, firms are said to generate validated learning (Shepherd & Gruber, 2021) upon which they base their subsequent business model decisions (Berends et al., 2021; Kirtley & O'Mahony, 2023; Ghezzi, 2019; Mansoori, 2017). The enactment of business model validation through experimentation has also been explicitly connected to widely popularized managerial approaches, such as the Lean Startup method (e.g., Bortolini et al., 2018; Contigiani & Levinthal, 2019; Hampel et al., 2020b; Mansoori, 2017; Shepherd & Gruber, 2021), Design Thinking (Klenner et al., 2022; Magistretti et al., 2022a; Mansoori & Lackeus, 2020), Design Sprint (Magistretti et al., 2022b), Growth Hacking (Troisi et al., 2020), and more.

As illustrated above, the existing body of literature is devoting growing and significant attention to the matter of business model validation. However, I contend that the current understanding of how new ventures perform business model validation has mostly been limited to individual experiences and decision-making (e.g., Camuffo et al., 2020; Grimes, 2018) or business model validation as a process (e.g., Contigiani & Levinthal, 2019; McDonald & Eisenhardt, 2020; Shepherd & Gruber, 2021; Silva et al., 2021). This interest has not been matched, however, with attention paid to how new ventures organize internally to carry out business model validation through experimentation.

Organizing at the early stages of an entrepreneurial venture or business model’s development, however, is one of the crucial challenges that firms encounter (Burton et al., 2019; Desantola & Gulati, 2017; Eisenmann & Wagonfeld, 2012). Looking at the current understanding of business model validation with a microfoundational lens (Felin et al., 2012, 2015), the current body of knowledge appears to lack a perspective on the structure of business model validation, intended as “the conditions that enable and constrain individual and collective action and establish the context for interactions within an organization” (Felin et al., 2012, p. 1364). In particular, previous studies fail to address how new ventures organize to conduct experimentation in business model validation. As a matter of fact, the issue of how new ventures should design their organizational structures altogether has until now failed to receive significant attention in the academic debate (Burton et al., 2019), despite the well-documented importance of the issue of organizing to guarantee venture survival (Desantola & Gulati, 2017). The current state of the art, indeed, seems to take for granted that new ventures are rudimentary versions of established organizations where crucial organizing issues–i.e., the division of labor and coordinating efforts–emerge spontaneously rather than as the result of deliberate design choices (Burton et al., 2019). In particular, the current literature addressed the issue of organizational design for top-management teams by comparing family vs non-family firms in corporate entrepreneurship programs (De Massis et al., 2021), and underlined the importance of designing open business models to keep communication open with the surrounding ecosystem for established firms (Fjeldstad & Snow, 2018), proposed that subordination through hierarchy–as opposed to organic coordination through heterarchy–may drive differences in a firm's stance when capitalizing on entrepreneurial opportunities (Berglund et al., 2020), or underlined the relevance of organizational choices in a new venture’s growth (DeSantola & Gulati, 2017). However, little is yet known about how new ventures structure themselves when carrying out experimentation to achieve business model validation.

### Business model scaling

Although several studies investigate the different facets of business model validation in both nascent ventures (Ghezzi & Cavallo, 2020; McDonald & Eisenhardt, 2020; Silva et al., 2021) and established firms (Chesbrough & Tucci, 2020; Hampel et al., 2020b), an emerging scholarly debate is raising the issue of understanding what happens after such validation is reached (Shepherd & Patzelt, 2022). As a matter of fact, once new ventures reach the validation of their business model, they need to capitalize on this newly-found awareness and direct their efforts toward *scaling* the business model (Eisenmann et al., 2012; Eisenmann, 2021a; Picken, 2017).

Business model scaling has been described in the context of digital new ventures to refer to the process of widening a new venture’s customer base (Busch & Barkema, 2022) without proportionate commitment in resource deployment (Huang et al., 2017) and capability development (Eisenmann & Wagonfeld, 2012). In other words, business model scaling means being able to achieve profitable growth by increasing the new venture’s user base while keeping the rest of its business model steady (Eisenmann & Wagonfeld, 2012; Huang et al., 2017; Nielsen & Lund, 2018). For example, as the UK-based scaleup Soldo reached a substantial customer base among well-established corporations with its employee expense management platform and prepaid cards, it started seeking new opportunities to increase its customer base. The venture soon launched an additional product–Soldo Drive, a card designed to specifically manage fuel expenses–that targeted individual professionals and exposed the scaleup to an entirely new market without having to develop additional specialized competences to serve it.

Scaling is vital to a new venture’s lifecycle (Picken, 2017) as it constitutes the natural evolution of its transition towards a full-rounded enterprise (Blank, 2013) and the development of its organizational identity (Snihur & Clarysse, 2022) as the venture collects additional information about the opportunity it is pursuing (Alexy et al., 2021). Business model scaling can happen through different means, such as saturating the existing target market by attacking the vast majority, addressing new customer segments, opening new distribution channels to reach a wider audience, or enriching the new venture’s offering to broaden its potential target market (Eisenmann, 2021a, b; Nielsen & Lund, 2018). However, choosing which segments to pursue first can mark the success (or failure) of a business model scaling process (Teece, 2018), and eventually of the entire new venture as failure to scale is often translated into a failure to survive (DeSantola & Gulati, 2017; Eisenmann, 2021b; Nielsen & Lund, 2018).

As this is one of the most threatening and crucial moments, especially for new ventures, in a business model’s lifecycle (Picken, 2017), business model scaling should be devoted careful attention. In this sense, building on Contigiani and Levinthal (2019), I argue that, despite the growing popularity of experimentation as a valid means to avoid new venture failure by anticipating market information, the vast majority of studies on experimentation address only the initial phases of a new venture’s lifecycle seeking business model validation (e.g., De Cock et al., 2020; McDonald & Eisenhardt, 2020), disregarding how experimentation may translate into business model scaling. Furthermore, as new ventures scale their business model, they face growing complexity and need to organize to make up for it (DeSantola & Gulati, 2017). New ventures face several issues related to scaling: keeping their focus, positioning their offering in the expanded market, building proper management, developing processes and infrastructures needed to run and scale the business, building a sustainable source of revenues, developing a culture that reflects the company's strategy, and managing vulnerabilities and risks that may be amplified when scaling (Picken, 2017). Organizing thus becomes a particularly critical matter for new ventures as they grow (DeSantola & Gulati, 2017), in that organizational design has also been demonstrated to influence their capability to exploit opportunities (De Massis et al., 2021). The emerging debate on scaling is pointing fingers at the largely disregarded matter of organizing for it, contending that the appropriate management of knowledge and the formalization of roles and procedures throughout the organization may facilitate successful scaling (Shepherd & Patzelt, 2022).

Thus, as business model scaling constitutes such a critical, yet complex phase for a new venture’s (or more generally, a new business model’s) lifecycle, I content that the current literature on business model scaling presents some relevant gaps. On the one hand, previous studies have yet seemingly failed to provide a clear understanding of how the process of business model scaling takes place, and whether and how experimentation may serve the purpose of enacting scaling as well as it served that of validation at earlier stages. On the other hand, the current understanding of business model scaling disregards the central theme of organizing for scaling and its direct consequences on how to manage knowledge transfer within a growing organization.

### Business model pivots

The term “pivot”–originally coined to indicate the basketball move where the player changes direction while keeping one leg steady to avoid wasting precious steps–was revamped by Eric Ries (2011), author of the book *The Lean Startup*, who transposed it to the management jargon to indicate a reorientation of the firm’s strategy despite not changing its long-term vision. In the Lean Startup approach (Blank, 2013; Ries, 2011), pivots are one among the array of potential decisions to be made as a result of the process of experimentation on the firm’s business model, as opposed to the decisions to persevere with the business model as-is, or alternatively perish and abandon the endeavor completely (Contigiani & Levinthal, 2019; Eisenmann et al., 2012; Frederiksen & Brem, 2017; Sanasi & Ghezzi, 2022).

Business model pivots thus embody the vehicle through which the learnings produced through experimentation drive shifts in trajectory from the current business model (Contigiani & Levinthal, 2019; Sala et al., 2021) destined to probe the business model’s underlying hypotheses (Ries, 2011). Previous studies agree that the nature of pivots is inherently experimental (Pillai et al., 2020), in that it enables firms to learn when their business model is no longer viable (Hampel et al., 2020a; McDonald & Gao, 2019), or when problems or new possibilities arise (Berends et al., 2021; Kirtley & O'Mahony, 2023) so that it needs fundamental alterations in its strategy, goals, technological applications, market focus, or identity (Berends et al., 2021; Camuffo et al., 2020; Contigiani & Levinthal, 2019; Hampel et al., 2020a; McDonald & Gao, 2019) to preserve competitive advantage (Snihur & Clarysse, 2022). The delivery platform Glovo, for example, when faced with the sudden sanitary restrictions and restaurant closings triggered by the COVID-19 pandemic, pivoted to a new value proposition by opening the so-called dark stores for the storage and delivery of groceries and other basic day-to-day products that encountered strong demand during the pandemic (Sanasi & Ghezzi, 2022).

Prior literature reports that pivots are one of the most common strategic decisions in new ventures (Flechas Chaparro & de Vasconcelos Gomes, 2021) in that they ensure the flexibility that is fundamental to cope with environmental uncertainty that characterizes nascent markets and new ventures (Zuzul & Tripsas, 2020). Pivot decisions have also been associated with a better likelihood of success (Pillai et al., 2020), as opposed to blind perseverance despite negative feedback (Camuffo et al., 2020). As a matter of fact, pivots occur as a response to unexpected events that compromise the viability of the firm’s current business model–such as when new opportunities or problems emerge (Berends et al., 2021; Kirtley & O'Mahony, 2023), or when entrepreneurs are faced with negative expert (Cohen et al., 2019; Grimes, 2018) or market feedback (Camuffo et al., 2020; McDonald & Gao, 2019; Sala et al., 2021)–so that the company can no longer guarantee the commitments it made in terms of timing and relationships with key stakeholders (Berends et al., 2021).

Previous studies also reported that pivots are only enacted in the circumstances of severe resource constraints and thus only concern new ventures (Hampel et al., 2020a), and force ventures to focus on short-term objectives (Berends et al., 2021). On the other hand, others argue that pivoting firms engage in paradoxical behavior (Gans et al., 2019) as pivoting requires them to irreversibly commit significant resources to the new strategic orientation (Pillai et al., 2020), missing out on the experimental nature of the pivot itself.

As business model pivots promise to enable firms to probe new hypotheses about their business model to restore its viability following an experimental fashion, they may serve as an important ally in particularly restrained situations, such as organizational crises and exogenous jolts. Previous studies have reported on the use of pivots as strategic responses to the COVID-19 crisis (Sanasi & Ghezzi, 2022). These accounts, however, are limited. Most of previous accounts on business model pivots had a definitory nature and has contributed to better defining the concept from a theoretical perspective (e.g., Berends et al., 2021; Kirtley & O'Mahony, 2023; Pillai et al., 2020). The literature on the antecedents and consequences of business model pivots in the context of different types of exogenous jolts or other types of idiosyncratic circumstances, on the other hand, is still widely underexplored.

## A future research agenda on entrepreneurial experimentation in business model dynamics

The previous sections provided an overview of the extant understanding of business model dynamics, proposing an original elaboration on the main theoretical concepts that constitute it while inquiring about their overlaps with the key concepts from entrepreneurial experimentation. To achieve this purpose, the review builds on the theoretical concepts presented in the previous sections to offer a critical stance toward the gaps that stem from each research stream identified and their intersection with the entrepreneurial experimentation literature. These research gaps give rise to a series of future research directions and research questions that may inform future scholarship in both entrepreneurship and strategy.

The research gaps identified, stemming from the theoretical concepts presented earlier, are listed in the following sections. At the end of each section, a set of sample research questions that may guide future research is presented. The research gaps and research questions formulated are summarized in Table 2.

**Table 2:** Summary of the proposed research agenda and related research questions

Research avenue	Research gaps	Illustrative research questions
*(1) Developing cumulative knowledge on entrepreneurial experimentation across business model dynamics*	The current body of literature lacks the necessary “cumulativeness” for theory building that adopts a phenomenological perspective in the study of business model dynamics Adopting a phenomenological perspective, business model dynamics could serve as a unit of analysis for the investigation of multiple evolutionary phenomena related to a firm’s strategy, as well as on the use of experimentation as their enactment. The literature is also disproportionately focused on selected business model dynamics (i.e., business model innovation)	• How can firms or other types of organizations conduct experimentation across business model dynamics? • What are the antecedents / consequences of experimentation across business model dynamics? • What are the contributions that a phenomenological view on business model dynamics can make to other research streams? • What are the boundary conditions of experimentation across business model dynamics?
*(2) Setting the boundary conditions of experimentation for business model innovation*	Firms encounter relevant barriers to experimentation which could significantly hinder their ability to introduce business model innovation, particularly in established firms (e.g., reputational threats). Extant literature identified the need to investigate the boundary conditions of business model innovation, even more so in contexts where significant barriers to experiment to achieve business model innovation are present, to spur theory building	• What are the boundary conditions of experimentation for business model innovation? • How do well-established firms carry out business model innovation through experimentation? • How do firms conduct business model innovation through experimentation in hyper-regulated markets? • What barriers to experimentation affect firm performance in introducing business model innovation?
*(3) Moving from process to structure in understanding experimentation for business model validation*	The current understanding of how new ventures perform business model validation has mostly been limited to individual experiences and decision-making or business model validation as a process. Adopting a microfoundational approach to business model validation, the current body of understanding lacks a perspective on how new ventures structure business model validation–i.e., how they organize for it. The current state of the art seems to take for granted that new ventures are rudimentary versions of established organizations where crucial organizing issues emerge spontaneously rather than as the result of deliberate design choices	• How do firms organize for experimentation to attain business model validation? • How does organizing for business model validation differ between established firms and new ventures? • What are the performance implications of experimentation for business model validation?
*(4) Extending experimentation to business model scaling*	Despite the growing popularity of experimentation as a valid means to avoid new venture failure by anticipating market information, the majority of the studies on experimentation address only the initial phases of a new venture’s lifecycle as it is seeking business model validation, disregarding how experimentation may translate to business model scaling As new ventures scale their business model, they face growing complexity and need to organize to make up for it. The emerging debate on scaling is pointing fingers to this largely disregarded matter of organizing for it. Scholars contend that the appropriate management of knowledge and the formalization of roles and procedures throughout the organization may facilitate successful scaling	• How can experimentation support conducting business model scaling in well-established firms / new ventures? • How can new ventures manage knowledge sharing as they scale their business model? • What are the factors that may influence successful business model scaling enacted through experimentation? • What are the performance implications of experimentation during scaling?
*(5) Investigating business model pivots as strategic responses to idiosyncratic conditions*	The current theoretical understanding on the nature of pivots of firms’ business models is fragmented as it lacks a characterization of pivots that reflects the temporary and experimental impression of the term. This issue becomes of utmost importance when firms are required to maintain high flexibility to face significant levels of uncertainty, like the ones characterizing contexts of crises, such as exogenous jolts or organizational crises. In these contexts, as well as in other settings characterized by restraining conditions, firms need to probe viable alternatives without causing irreparable damage and potentially compromising their own existence	• How can firms deploy pivots in the context of constrained conditions? • How can firms deploy pivots as a response to unfolding organizational crises? • What are the performance implications of deploying pivots as a response to exogenous jolts as opposed to ordinary circumstances? • How do firms manage legitimacy issues related to pivoting during exogenous jolts / organizational crises?

### Research avenue 1: Developing cumulative knowledge on entrepreneurial experimentation across business model dynamics

As presented in the previous sections of this review, recent studies suggest that the view of the business model as a static picture of the logic of a firm may be the source of the doubts arising in current literature, arguing it only represents “strategy in new bottles” (e.g., Bigelow & Barney, 2021; Massa et al., 2017). In response to this critique, multiple authors have advanced the concept of business model dynamics (Foss & Saebi, 2018), proposing to view the business model as a dynamic entity that encompasses the alterations and developments in the firm’s strategy (Achtenhagen et al., 2013; Demil & Lecocq, 2010; Foss & Saebi, 2017, 2018). This way, business model dynamics promise to grant the business model concept legitimacy and elevates the business model as the unit of analysis for evolutionary phenomena taking place within firms (Demil & Lecocq, 2010; Foss & Saebi, 2018).

However, past efforts to review the extant body of literature on business model dynamics have mostly been plagued by a lack of cumulativeness in driving a univocal understanding of what business model dynamics meant (Foss & Saebi, 2018), witnessing disproportionate attention paid to business model innovation as compared to other business model dynamics (Foss & Saebi, 2017). This lack of cumulativeness stems from the lack of investigation of what the literature on business model dynamics is built upon from a theoretical standpoint, as well as the unclear relationship and common misunderstanding between different business model dynamics. Building on these arguments, future research may hence devote efforts in substantiating the current scholarly understanding of the use of entrepreneurial experimentation in business model dynamics. Adopting this phenomenological lens, future studies may observe business model dynamics as a unit of analysis to interpret diverse evolutionary phenomena related to a firm’s strategy, setting the stage for building the cumulativeness needed for the research stream to set off (Foss & Saebi, 2018). Thus, the following research agenda may provide promising avenues for bridging these emerging literature gaps.

- How can firms or other types of organizations conduct experimentation across business model dynamics?
- What are the antecedents / consequences of experimentation across business model dynamics?
- What are the boundary conditions of experimentation across business model dynamics?
- What are the contributions that a phenomenological view on business model dynamics can make to other research streams?

### Research avenue 2: Setting the boundary conditions of experimentation for business model innovation

The most widely studied among business model dynamics is undoubtedly business model innovation (e.g., Casadesus-Masanell & Zhu, 2013; Schneckenberg et al., 2022; Zott et al., 2011). Yet, although the growing body of literature devoted to it underlines the increasing relevance given to its investigation as a self-standing phenomenon, it is still lacking cumulativeness, hindering theory building (Foss & Saebi, 2017, 2018). Attempts to provide one single unifying model to systematize its multilevel and multidimensional nature have so far been mostly unsuccessful (Massa et al., 2017; Schneckenberg et al., 2022). Furthermore, the theorizing efforts for business model innovation lack one fundamental aspect. Besides discussing what business model innovation represents, how it is enacted through experimentation and why, the current scholarly understanding lacks the definition of its boundary conditions (Foss & Saebi, 2017)–namely the “who, where, when” (Whetten, 1989). However, given that theories in social sciences cannot exist irrespective of the context they are observed in, and thus generalizable to (Busse et al., 2017), the definition of boundary conditions in theorizing in the social sciences acquires particular relevance. Thus, the boundary conditions of business model innovation should depict the accuracy of its predictions in a given empirical context (Busse et al., 2017). Supported by previous studies, which underlined the hurdles of carrying out business model innovation in established firms (Chesbrough, 2010; Foss & Saebi, 2017) and how experimentation may be hindered by their characteristics such as, for example, how firms are viewed by their key stakeholders (Chesbrough & Tucci, 2020; Hampel et al., 2020b), this review raises and highlights this relevant gap. In particular, an emerging body of research starting to investigate the idiosyncratic context of experimentation for business model innovation in high-reputation firms (Sanasi et al., 2022), identifying the importance of de-risking core assumptions of the newly implemented business model by adopting value propositions that were already validated by others–following a copycat logic that is common among new ventures (Frankenberger & Stam, 2020). However, future research may broaden the scope of the inquiry related to the boundary conditions to experimentation for business model innovation, expanding the scope of these considerations to broader or currently unexplored settings (e.g., hyper-regulated markets (Magistretti et al., 2021)). Future studies may address, among others, how other types of constraints may affect the way experimentation is carried out in idiosyncratic endeavors that may involve the presence of barriers to experimentation in the introduction of business model innovation. The following set of research questions therefore suggests an illustrative future research agenda to expand the body of literature on experimentation in business model innovation and develop more thorough understanding of its boundary conditions.

- What are the boundary conditions of experimentation for business model innovation?
- How do well-established firms carry out business model innovation through experimentation?
- How do firms conduct business model innovation through experimentation in hyper-regulated markets?
- What barriers to experimentation affect firm performance in introducing business model innovation?

### Research avenue 3: Moving from individual and process to structure in understanding experimentation for business model validation

As business model validation is gaining ground in the management literature as a fundamental process, especially for early-stage new ventures, to assess the viability of a business model’s underlying hypotheses (Ghezzi & Cavallo, 2020; McDonald & Eisenhardt, 2020; Shepherd & Gruber, 2021). Several accounts have acknowledged that this process is carried out through experimentation (e.g., Camuffo et al., 2020; Contigiani & Levinthal, 2019) and have investigated how this process reflects upon individual experiences and decision-making (e.g., Grimes, 2018). However, adopting a microfoundational lens (Felin et al., 2012, 2015) to examine the current understanding of the phenomenon, previous studies only addressed the individual and process dimensions of business model validation. This interest has not yet been matched with studies dedicated to investigating the structural dimension of experimentation for business model validation, intended as “the conditions that enable and constrain individual and collective action and establish the context for interactions within an organization” (Felin et al., 2012, p. 1364).

As a matter of fact, the scholarly understanding of how new ventures should design their organizational structures per se is still in its infancy: although a relevant matter for the success and survival of new ventures (DeSantola & Gulati, 2017), previous studies often picture new ventures as rudimentary versions of established companies, whose structural choices are emergent, rather than deliberate (Burton et al., 2019). Only a handful of studies are starting to hint at how new ventures structure processes as they grow may require dedicated research in the management and entrepreneurship literature (e.g., DeSantola & Gulati, 2017; Shepherd & Patzelt, 2022). However, little is yet known about how new ventures structure themselves when carrying out experimentation to achieve business model validation. The following research questions thus encourage future research to address and investigate this gap, so as to contribute to the ongoing scholarly discourse.

- How do firms organize for experimentation to attain business model validation?
- How does organizing for business model validation differ between established firms and new ventures?
- What are the performance implications of experimentation for business model validation?

### Research avenue 4: Extending experimentation to business model scaling

Despite the growing popularity of experimentation as a valid means to avoid new venture failure by anticipating market information (Agrawal et al*.*, 2021; McGrath, 1999), the vast majority of studies on experimentation address only the initial phases of a new venture’s lifecycle while seeking market validation (e.g., De Cock et al., 2020; McDonald & Eisenhardt, 2020) as they seek business model validation. This way, the majority of studies disregard how experimentation translates to the subsequent phase and what happens when the new venture is required to devote efforts to scaling its now-validated business model, once it has reached market validation (Contigiani & Levinthal, 2019; Picken, 2017). As a matter of fact, enduring resource scarcity during scaling (DeSantola & Gulati, 2017) and the necessity to increase the user base without proportionate resource commitments (Huang et al., 2017) would intuitively call for extensive use of experimentation also when attempting to scale the business model.

Furthermore, as new ventures scale their business model, they face growing complexity and need to organize to properly cope with it (DeSantola & Gulati, 2017). The emerging debate on scaling is pointing fingers at the largely disregarded matter of organizing in the context of scaling (Shepherd & Patzelt, 2022). In particular, scholars contend that the appropriate management of knowledge and the formalization of roles and procedures throughout the organization are of utmost importance when scaling (Shepherd & Patzelt, 2022). Therefore, this review encourages future research to extend the scholarly understanding of business model scaling by investigating the way new ventures experiment after they have reached market validation, as well as by trying to shed light on the mechanisms they set in place to enable experimentation at an organizational level despite the growing complexity that comes with scaling. The above considerations led us to formulate the following set of illustrative research questions, that may inspire future research in this direction:

- How can experimentation support the process of business model scaling in well-established firms / new ventures?
- How can new ventures manage knowledge sharing as they scale their business model?
- What are the factors that may influence successful business model scaling enacted through experimentation?
- What are the performance implications of experimentation during scaling?

### Research avenue 5: The antecedents and consequences of business model pivots

Due to the recent advent of the worldwide emergency triggered by the COVID-19 pandemic, firms, and in particular small businesses and new ventures (Caiazza et al., 2021), were hardly hit by the restrictive measures on movement and social distancing imposed to contain the outbreaks, including extensive lockdowns in wide geographical areas and had to come up with strategic responses to face the crisis. In particular, this dramatically constraining situation crisis led multiple firms to pivot their business model to better align with the unfolding external events and responde to rapidly changing environmental conditions (Sanasi & Ghezzi, 2022).

As the crisis is threatening the survival of companies across the globe (Wenzel et al*.*, 2021), a growing body of accounts from the strategic management and entrepreneurship literature has urged research to investigate how to stem the enormous consequences of crises and the way they impact the strategy-making processes of both new and established ventures (e.g., Dushnitsky et al., 2020; Björklund et al*.*, 2020; Foss and Klein, 2020; Giones et al*.*, 2020; Klein, 2020; Kuckertz et al., 2020; Newman et al., 2022; Shepherd, 2020; Thorgren & Williams, 2020). In this context, multiple authors are calling for a revision of some of the current assumptions in management research (De Massis & Rondi, 2020; Shepherd, 2020) as well as raising a quest for investigating newly emerging research questions (e.g., Dushnitsky et al., 2020; Shepherd & Williams, 2020).

On the other hand, entrepreneurship scholars have devoted significant attention to how new ventures reduce the uncertainty connected to their endeavors. New ventures are said to engage in experimentation to gather information and reduce the uncertainty about the viability of their business models (Andries & Debackere, 2007; Andries et al., 2013). When business model viability is not verified by the experiments or compromised by the emergence of an unexpected event, new ventures are said to pivot (Berends et al., 2021; Kirtley & O'Mahony, 2023), recombining existing resources to accomplish a new strategic orientation (Hampel et al., 2020a; McDonald & Gao, 2019).

Crises compromise the viability of the business models of both established firms and new ventures (Miklian & Hoelscher, 2021; Newman et al., 2022) and require them to formulate swift responses (Pearson & Clair, 1998) by recombining the resources they have at hand to face the rising uncertainty (Klein, 2020; Rindova & Kotha, 2001). Crises can occur both as events, leading to sudden impacts and consequences for the viability of the business, or as longer, sometimes endogenous, development processes that take place within and across firms (Williams et al., 2017). While previous research has mainly looked at the impact of exogenous jolts by adopting a macro-perspective (Newman et al., 2022), leveraging the business model as a dynamic unit of analysis to examine firms’ responses to different kinds of crises–such as organizational crises and exogenous jolts–could contribute to informing the micro-perspective on strategic responses to crises and responding to emerging calls for this need (Newman et al., 2022). In this context, business model pivots can support firms with a tool that is specifically designed to probe new hypotheses on the firm’s business model (Frederiksen & Brem, 2017) through limited resource commitments (Hampel et al., 2020a), embodying vehicles of experimentation that reduce the extreme uncertainty carried by the crisis (Klein, 2020), turning adversity into opportunity (Andries et al*.*, 2020; Salvato et al., 2020). In fact, emerging research is beginning to look at how crises shape new ventures’ business models (Guckenbiehl & Corral de Zubielqui, 2022). Previous studies have suggested that pivots may provide a strategic response to situations of crisis (Sanasi & Ghezzi, 2022), while also suggesting that certain strategic decisions in new ventures–such as resource reconfiguration and seeking to regain environmental fit–may provide positive consequences for firm performance in the aftermath of an environmental jolt (Colombo et al., 2021). However, this area of research is still underexplored, and requires more extensive investigation and theorization in the context of business model pivots and entrepreneurial experimentation.

To better understand how the concept of pivot and its enactment through entrepreneurial experimentation can be transposed from the context of “ordinary” circumstances in entrepreneurship to that of responses to organizational crises, exogenous jolts and, more generally, idiosyncratic circumstances, future research may investigate research questions that contextualize business model pivots within these circumstances, as well as inquire the performance implications of deploying pivots on a firm’s business model within specific (as opposed to ordinary) conditions. Furthermore, as the literature on pivots has widely debated the legitimacy and identity issues connected to pivoting (e.g., Hampel et al., 2020a; McDonald & Gao, 2019), future research may investigate how legitimacy issues are managed in the context of repentine pivots as a response to exogenous jolts, such as crises. The following set of illustrative research questions are hence proposed to spur future research in this direction:

- How can firms deploy pivots in the context of constrained conditions?
- How can firms deploy pivots as a response to unfolding organizational crises?
- What are the performance implications of deploying pivots as a response to exogenous jolts as opposed to ordinary circumstances?
- How do firms manage legitimacy issues related to pivoting during exogenous jolts / organizational crises?

## Conclusion

This study provides an overview of the state-of-the-art understanding of business model dynamics, a concept that has been gaining momentum in the academic literature to refer to all the alterations to a firm's business model that enable it to produce sustained value creation throughout time, such as the developmental or change processes taking place in both entrepreneurial and incumbent firms. In particular, the study taps into the emerging shift in the current scholarly debate that argues against the traditional ontological stance in research on the business model concept as the realization of a firm’s strategy, in favor of a new phenomenological stance that views the business model as a unit of analysis for different phenomena related to the firm’s strategy. This shift gave rise to the concept of business model dynamics, whose inherently uncertain nature called for a parallel with emerging theory in entrepreneurship that looks at experimentation as the means to cope with uncertainty and enact business model dynamics.

This study thereby extends and consolidates the current scholarly understanding of entrepreneurial experimentation in and across business model dynamics. This view provided fertile ground for contributing to the ongoing discourse with an original theoretical interpretive framework, giving rise to an overview of the existing, as well as the identification of emerging gaps and promising future research avenues for seemingly interchangeable although distinct phenomena. This article presented the phenomena making up the different business model dynamics reported in previous literature as business model innovation, validation, scaling, and pivots, and discussed each individual dynamic’s relationship with entrepreneurial experimentation. Building on this newly found perspective, the current research gaps were illustrated and addressed through some illustrative research questions that may inform future research in the area. I am hopeful this review will stimulate future theoretical and empirical studies that wish to contribute to the literature on business model dynamics, providing scholars with a renewed and systematic understanding of different business model dynamics. On the other hand, by highlighting the emerging gaps in the overlap with entrepreneurial experimentation, this review opened the way for future contributions to try and address those gaps, or develop further research questions related to this domain.

### Limitations

This study is certainly not free from limitations. As business model dynamics is a complex and evolving phenomenon, this study has no ambition to comprehensively provide a taxonomy of business model dynamics enacted through entrepreneurial experimentation. Rather, this study wishes to extend the current scholarly understanding of entrepreneurial experimentation in and across business model dynamics and identify an overarching reference framework and research agenda.

In this sense, this review provides an interpretive and narrative overview of the literature on business model dynamics and its overlaps with entrepreneurial experimentation. However, future research may consider such a relationship systematically, narrowing down the selection of papers included in the review to those that only explicitly refer to both concepts simultaneously. Furthermore, consistently with the future research questions presented in the previous sections, future empirical research may tackle this study's perspective from an empirical standpoint. In this sense, empirical studies may not only investigate the relationship between business model dynamics and entrepreneurial experimentation in the different contexts identified but may potentially investigate their relationships with firm performance. Lastly, the studies included in this review predominantly address the phenomenon in for-profit established firms and entrepreneurial ventures. Although a limited number of studies has looked at the enactment of business model dynamics through experimentation in not-for-profit or informal organizations in the past (e.g., Sosna et al., 2010), this research directions certainly deserves dedicated and more thorough attention.

### Practical implications

This review can also provide fruitful contributions to practice. In particular, this study taps into practitioners’ interest in entrepreneurial experimentation to enact different business model dynamics, ignited in the latest years by the popularity of managerial approaches such as “the Lean Startup” method (Ries, 2011). This way, this study may trigger managerial and entrepreneurial curiosity by offering insights on the implementation of entrepreneurial experimentation to enact different business model dynamics within firms. In particular, the review identifies four distinct business model dynamics–namely business model innovation, validation, scaling, and pivots–that have been discussed in the existing management literature and offers an overview of the existing understanding on each. In this sense, managers and entrepreneurs may find a useful summary and systematization of the different business model dynamics they may encounter when having to innovate their venture’s strategy, validate it, scale it, or pivot. Furthermore, the study examines the current understanding of each business model dynamic and proposes emerging gaps that may be reserved future scholarly attention. This way, this review calls for further contributions which may fill gaps that can inform relevant issues encountered by the practice community. I hope this study may pave the way for both theory and practice to enrich such understanding, igniting the debate on business model dynamics and the way they are enacted through entrepreneurial experimentation.

## Footnotes

1 Academic Journal Guide of the Chartered Association of Business Schools https://charteredabs.org/academic-journal-guide-2021/

## References

[CR1] Achtenhagen L, Melin L, Naldi L (2013). Dynamics of business models–strategizing, critical capabilities and activities for sustained value creation. Long Range Planning.

[CR2] Agrawal A, Gans JS, Stern S (2021). Enabling entrepreneurial choice. Management Science.

[CR3] Alexy O, Poetz K, Puranam P, Reitzig M (2021). Adaptation or persistence? Emergence and revision of organization designs in new ventures. Organization Science.

[CR4] Andries P, Debackere K (2007). Adaptation and performance in new businesses: Understanding the moderating effects of independence and industry. Small Business Economics.

[CR5] Andries P, Debackere K, Van Looy B (2013). Simultaneous experimentation as a learning strategy: Business model development under uncertainty. Strategic Entrepreneurship Journal.

[CR6] Andries P, Debackere K, Van Looy B (2020). Simultaneous experimentation as a learning strategy: Business model development under uncertainty —Relevance in times of COVID-19 and beyond. Strategic Entrepreneurship Journal.

[CR7] Baker T, Nelson RE (2005). Creating something from nothing: Resource construction through entrepreneurial bricolage. Administrative Science Quarterly.

[CR8] Berends H, Smits A, Reymen I, Podoynitsyna K (2016). Learning while (re) configuring: Business model innovation processes in established firms. Strategic Organization.

[CR9] Berends H, van Burg E, Garud R (2021). Pivoting or persevering with venture ideas: Recalibrating temporal commitments. Journal of Business Venturing.

[CR10] Berglund H, Bousfiha M, Mansoori Y (2020). Opportunities as artifacts and entrepreneurship as design. Academy of Management Review.

[CR11] Bigelow LS, Barney JB (2021). What can strategy learn from the business model approach?. Journal of Management Studies.

[CR12] Björklund TA, Mikkonen M, Mattila P (2020). Expanding entrepreneurial solution spaces in times of crisis: Business model experimentation amongst packaged food and beverage ventures. Journal of Business Venturing Insights.

[CR13] Blank S (2013). Why the Lean start-up changes everything. Harvard Business Review.

[CR14] Bocken N, Snihur Y (2020). Lean Startup and the business model: Experimenting for novelty and impact. Long Range Planning.

[CR15] Bojovic N, Genet C, Sabatier V (2018). Learning, signaling, and convincing: The role of experimentation in the business modeling process. Long Range Planning.

[CR16] Bortolini RF, Cortimiglia MN, Danilevicz ADMF, Ghezzi A (2018). Lean startup: A comprehensive historical review. Management Decision.

[CR17] Breslin D, Gatrell C (2023). Theorizing through literature reviews: The miner-prospector continuum. Organizational Research Methods.

[CR18] Brown SL, Eisenhardt KM (1997). The art of continuous change: Linking complexity theory and time-paced evolution in relentlessly shifting organizations. Administrative Science Quarterly.

[CR19] Burton MD, Colombo MG, Rossi-Lamastra C, Wasserman N (2019). The organizational design of entrepreneurial ventures. Strategic Entrepreneurship Journal.

[CR20] Busch C, Barkema H (2022). Planned luck: How incubators can facilitate serendipity for nascent entrepreneurs through fostering network embeddedness. Entrepreneurship Theory and Practice.

[CR21] Busse C, Kach AP, Wagner SM (2017). Boundary conditions: What they are, how to explore them, why we need them, and when to consider them. Organizational Research Methods.

[CR22] Caiazza R, Phan P, Lehmann E, Etzkowitz H (2021). An absorptive capacity-based systems view of COVID-19 in the small business economy. International Entrepreneurship and Management Journal.

[CR23] Camuffo A, Cordova A, Gambardella A, Spina C (2020). A scientific approach to entrepreneurial decision making: Evidence from a randomized control trial. Management Science.

[CR24] Casadesus-Masanell R, Ricart JE (2010). From strategy to business models and onto tactics. Long Range Planning.

[CR25] Casadesus-Masanell R, Zhu F (2013). Business model innovation and competitive imitation: The case of sponsor-based business models. Strategic Management Journal.

[CR26] Cavallo A, Ghezzi A, Balocco R (2019). Entrepreneurial ecosystem research: Present debates and future directions. International Entrepreneurship and Management Journal.

[CR27] Chesbrough H (2004). Managing open innovation. Research-Technology Management.

[CR28] Chesbrough H (2010). Business model innovation: Opportunities and barriers. Long Range Planning.

[CR29] Chesbrough H, Rosenbloom RS (2002). The role of the business model in capturing value from innovation: Evidence from Xerox Corporation’s technology spin-off companies. Industrial and Corporate Change.

[CR30] Chesbrough H, Tucci CL (2020). The interplay between open innovation and lean startup, or, why large companies are not large versions of startups. Strategic Management Review.

[CR31] Cohen SL, Bingham CB, Hallen BL (2019). The role of accelerator designs in mitigating bounded rationality in new ventures. Administrative Science Quarterly.

[CR32] Colombo MG, Piva E, Quas A, Rossi-Lamastra C (2021). Dynamic capabilities and high-tech entrepreneurial ventures’ performance in the aftermath of an environmental jolt. Long Range Planning.

[CR33] Contigiani A, Levinthal DA (2019). Situating the construct of lean start-up: Adjacent conversations and possible future directions. Industrial and Corporate Change.

[CR34] Cortimiglia MN, Ghezzi A, Frank AG (2016). Business model innovation and strategy making nexus: Evidence from a cross-industry mixed-methods study. R&D Management.

[CR35] Cronin, M. A., & George, E. (2020). The why and how of the integrative review. *Organizational Research Methods*, in press.

[CR36] Cronin MA, George E (2023). The why and how of the integrative review. Organizational Research Methods.

[CR37] De Cock R, Bruneel J, Bobelyn A (2020). Making the lean start-up method work: The role of prior market knowledge. Journal of Small Business Management.

[CR38] De Massis AV, Rondi E (2020). COVID-19 and the future of family business research. Journal of Management Studies.

[CR39] De Massis A, Eddleston KA, Rovelli P (2021). Entrepreneurial by design: How organizational design affects family and non-family firms’ opportunity exploitation. Journal of Management Studies.

[CR40] Demil B, Lecocq X (2010). Business model evolution: In search of dynamic consistency. Long Range Planning.

[CR41] Demil B, Lecocq X, Ricart JE, Zott C (2015). Introduction to the SEJ special issue on business models: Business models within the domain of strategic entrepreneurship. Strategic Entrepreneurship Journal.

[CR42] DeSantola A, Gulati R (2017). Scaling: Organizing and growth in entrepreneurial ventures. Academy of Management Annals.

[CR43] Doganova L, Eyquem-Renault M (2009). What do business models do?: Innovation devices in technology entrepreneurship. Research Policy.

[CR44] Doz YL, Kosonen M (2010). Embedding strategic agility: A leadership agenda for accelerating business model renewal. Long Range Planning.

[CR45] Dushnitsky G, Graebner M, Zott C (2020). Entrepreneurial responses to crisis. Strategic Entrepreneurship Journal.

[CR46] Ehrig T, Foss NJ (2022). Unknown unknowns and the treatment of firm-level adaptation in strategic management research. Strategic Management Review.

[CR47] Eisenmann TR (2021). Why startups fail: A new roadmap for entrepreneurial success.

[CR48] Eisenmann TR (2021). Why startups fail. Harvard Business Review.

[CR49] Eisenmann, T. R., & Wagonfeld, A. B. (2012). Scaling a startup: People and organizational issues. *Harvard Business School Entrepreneurial Management Case*, 812–100.

[CR50] Eisenmann, T. R., Ries, E., & Dillard, S. (2012). Hypothesis-driven entrepreneurship: The lean startup. *Harvard Business School Entrepreneurial Management Case*, 812–095.

[CR51] Felin T, Zenger TR (2009). Entrepreneurs as theorists: On the origins of collective beliefs and novel strategies. Strategic Entrepreneurship Journal.

[CR52] Felin T, Zenger TR (2017). The theory-based view: Economic actors as theorists. Strategy Science.

[CR53] Felin T, Foss NJ, Heimeriks KH, Madsen TL (2012). Microfoundations of routines and capabilities: Individuals, processes, and structure. Journal of Management Studies.

[CR54] Felin T, Foss NJ, Ployhart RE (2015). The microfoundations movement in strategy and organization theory. Academy of Management Annals.

[CR55] Felin, T., Gambardella, A., Stern, S., & Zenger, T. (2019). Lean startup and the business model: Experimentation revisited. *Forthcoming in Long Range Planning.*

[CR56] Felin T, Gambardella A, Stern S, Zenger T (2020). Lean startup and the business model: Experimentation revisited. Long Range Planning.

[CR57] Fjeldstad ØD, Snow CC (2018). Business models and organization design. Long Range Planning.

[CR58] Flechas Chaparro XA, de Vasconcelos Gomes LA (2021). Pivot decisions in startups: a systematic literature review. International Journal of Entrepreneurial Behavior & Research.

[CR59] Foss NJ, Klein PG (2020). Entrepreneurial opportunities: Who needs them?. Academy of Management Perspectives.

[CR60] Foss NJ, Saebi T (2017). Fifteen years of research on business model innovation: How far have we come, and where should we go?. Journal of Management.

[CR61] Foss NJ, Saebi T (2018). Business models and business model innovation: Between wicked and paradigmatic problems. Long Range Planning.

[CR62] Frankenberger K, Stam W (2020). Entrepreneurial copycats: A resource orchestration perspective on the link between extra industry business model imitation and new venture growth. Long Range Planning.

[CR63] Frederiksen DL, Brem A (2017). How do entrepreneurs think they create value? A scientific reflection of Eric Ries’ Lean Startup approach. International Entrepreneurship and Management Journal.

[CR64] Gambardella A, McGahan AM (2010). Business-model innovation: General purpose technologies and their implications for industry structure. Long Range Planning.

[CR65] Gans JS, Stern S, Wu J (2019). Foundations of entrepreneurial strategy. Strategic Management Journal.

[CR66] Garud R, Karunakaran A (2018). Process-based ideology of participative experimentation to foster identity-challenging innovations: The case of Gmail and AdSense. Strategic Organization.

[CR67] Ghezzi A (2019). Digital startups and the adoption and implementation of lean startup approaches: Effectuation, bricolage and opportunity creation in practice. Technological Forecasting and Social Change.

[CR68] Ghezzi A (2020). How Entrepreneurs make sense of Lean Startup Approaches: Business Models as cognitive lenses to generate fast and frugal Heuristics. Technological Forecasting and Social Change.

[CR69] Ghezzi A, Cavallo A (2020). Agile business model innovation in digital entrepreneurship: Lean startup approaches. Journal of Business Research.

[CR70] Giones F, Brem A, Pollack JM (2020). Revising entrepreneurial action in response to exogenous shocks: Considering the COVID-19 pandemic. Journal of Business Venturing Insights.

[CR71] Grimes MG (2018). The pivot: How founders respond to feedback through idea and identity work. Academy of Management Journal.

[CR72] Guckenbiehl P, Corral de Zubielqui G (2022). Start-ups’ business model changes during the COVID-19 pandemic: Counteracting adversities and pursuing opportunities. International Small Business Journal.

[CR73] Hampel C, Perkmann M, Phillips N (2020). Beyond the lean start-up: Experimentation in corporate entrepreneurship and innovation. Innovation: Organization and Management.

[CR74] Hampel CE, Tracey P, Weber K (2020). The art of the pivot: How new ventures manage identification relationships with stakeholders as they change direction. Academy of Management Journal.

[CR75] Huang J, Henfridsson O, Liu MJ, Newell S (2017). Growing on steroids: Rapidly scaling the user base of digital ventures through digital innovation. MIS Quarterly.

[CR76] Katila R, Shane S (2005). When does lack of resources make new firms innovative?. Academy of Management Journal.

[CR77] Kerr WR, Nanda R, Rhodes-Kropf M (2014). Entrepreneurship as experimentation. Journal of Economic Perspectives.

[CR78] Kirtley J, O'Mahony S (2023). What is a pivot? Explaining when and how entrepreneurial firms decide to make strategic change and pivot. Strategic Management Journal.

[CR79] Klein PG (2020). Uncertainty and entrepreneurial judgment during a health crisis. Strategic Entrepreneurship Journal.

[CR80] Klenner NF, Gemser G, Karpen IO (2022). Entrepreneurial ways of designing and designerly ways of entrepreneuring: Exploring the relationship between design thinking and effectuation theory. Journal of Product Innovation Management.

[CR81] Kuckertz A, Brändle L, Gaudig A, Hinderer S, Reyes CAM, Prochotta A, Berger ES (2020). Startups in times of crisis–A rapid response to the COVID-19 pandemic. Journal of Business Venturing Insights.

[CR82] Lanzolla G, Markides C (2021). A business model view of strategy. Journal of Management Studies.

[CR83] Leatherbee M, Katila R (2020). The lean startup method: Early-stage teams and hypothesis-based probing of business ideas. Strategic Entrepreneurship Journal.

[CR84] Lichtenstein BB, Carter NM, Dooley KJ, Gartner WB (2007). Complexity dynamics of nascent entrepreneurship. Journal of Business Venturing.

[CR85] Loch CH, Solt ME, Bailey EM (2008). Diagnosing unforeseeable uncertainty in a new venture. Journal of Product Innovation Management.

[CR86] Magistretti S, Allo L, Verganti R, Dell’Era C, Reutter F (2021). The microfoundations of design sprint: How Johnson & Johnson cultivates innovation in a highly regulated market. Journal of Knowledge Management.

[CR87] Magistretti S, Dell’Era C, Verganti R, Bianchi M (2022). The contribution of design thinking to the R of R&D in technological innovation. R&D Management.

[CR88] Magistretti, S., Sanasi, S., Dell'Era, C., & Ghezzi, A. (2022b). Entrepreneurship as design: A design process for the emergence and development of entrepreneurial opportunities. *Creativity and Innovation Management*, in press. 10.1111/caim.12529

[CR89] Magretta J (2002). Why business models matter. Harvard Business Review.

[CR90] Mansoori Y (2017). Enacting the lean startup methodology: The role of vicarious and experiential learning processes. International Journal of Entrepreneurial Behavior & Research.

[CR91] Mansoori Y, Lackeus M (2020). Comparing effectuation to discovery-driven planning, prescriptive entrepreneurship, business planning, lean startup, and design thinking. Small Business Economics.

[CR92] Massa L, Tucci CL, Afuah A (2017). A critical assessment of business model research. Academy of Management Annals.

[CR93] McDonald RM, Eisenhardt KM (2020). Parallel play: Startups, nascent markets, and effective business-model design. Administrative Science Quarterly.

[CR94] McDonald R, Gao C (2019). Pivoting isn’t enough? Managing strategic reorientation in new ventures. Organization Science.

[CR95] McGrath RG (1999). Falling forward: Real options reasoning and entrepreneurial failure. Academy of Management Review.

[CR96] McGrath RG (2010). Business models: A discovery driven approach. Long Range Planning.

[CR97] McGrath, R. G., & MacMillan, I. C. (1995). Discovery-Driven Planning. *Harvard Business Review, 73*(4).

[CR98] Miklian J, Hoelscher K (2021). SMEs and exogenous shocks: A conceptual literature review and forward research agenda. International Small Business Journal.

[CR99] Murray F, Tripsas M (2004). The exploratory processes of entrepreneurial firms: The role of purposeful experimentation. Advances in Strategic Management.

[CR100] Nambisan S (2017). Digital entrepreneurship: Toward a digital technology perspective of entrepreneurship. Entrepreneurship Theory and Practice.

[CR101] Newman A, Obschonka M, Block J (2022). Small businesses and entrepreneurship in times of crises: The renaissance of entrepreneur-focused micro perspectives. International Small Business Journal.

[CR102] Nielsen C, Lund M (2018). Building scalable business models. MIT Sloan Management Review.

[CR103] Oliva R, Kallenberg R (2003). Managing the transition from products to services. International Journal of Service Industry Management.

[CR104] Pearson CM, Clair JA (1998). Reframing crisis management. Academy of Management Review.

[CR105] Picken JC (2017). From startup to scalable enterprise: Laying the foundation. Business Horizons.

[CR106] Pillai SD, Goldfarb B, Kirsch DA (2020). The origins of firm strategy: Learning by economic experimentation and strategic pivots in the early automobile industry. Strategic Management Journal.

[CR107] Popper KR (1963). Science as falsification. Conjectures and Refutations.

[CR108] Porter ME (1996). What is strategy. Harvard Business Review.

[CR109] Post C, Sarala R, Gatrell C, Prescott JE (2020). Advancing theory with review articles. Journal of Management Studies.

[CR110] Priem RL, Wenzel M, Koch J (2018). Demand-side strategy and business models: Putting value creation for consumers center stage. Long Range Planning.

[CR111] Ries E (2011). The lean startup: How today’s entrepreneurs use continuous innovation to create radically successful businesses.

[CR112] Rindova V, Courtney H (2020). To shape or adapt: Knowledge problems, epistemologies, and strategic postures under Knightian uncertainty. Academy of Management Review.

[CR113] Rindova VP, Kotha S (2001). Continuous “morphing”: Competing through dynamic capabilities, form, and function. Academy of Management Journal.

[CR114] Sala PK, Philbin SP, Barikzai S (2021). A qualitative research study of the tech startup journey through entrepreneurial pivoting. International Journal of Entrepreneurial Behavior & Research.

[CR115] Salvato C, Sargiacomo M, Amore MD, Minichilli A (2020). Natural disasters as a source of entrepreneurial opportunity: Family business resilience after an earthquake. Strategic Entrepreneurship Journal.

[CR116] Sanasi, S., & Ghezzi, A. (2022). Pivots as strategic responses to crises: Evidence from Italian companies navigating COVID-19. *Strategic Organization*, in press. 10.1177/14761270221122933

[CR117] Sanasi S, Ghezzi A, Cavallo A, Rangone A (2020). Making sense of the sharing economy: A business model innovation perspective. Technology Analysis & Strategic Management.

[CR118] Sanasi S, Manotti J, Ghezzi A (2022). Achieving agility in high-reputation firms: Agile experimentation revisited. IEEE Transactions on Engineering Management.

[CR119] Sarasvathy SD (2001). Causation and effectuation: Toward a theoretical shift from economic inevitability to entrepreneurial contingency. Academy of Management Review.

[CR120] Schneckenberg D, Matzler K, Spieth P (2022). Theorizing business model innovation: an organizing framework of research dimensions and future perspectives. R&D Management.

[CR121] Shepherd DA (2020). COVID 19 and entrepreneurship: Time to pivot?. Journal of Management Studies.

[CR122] Shepherd DA, Gruber M (2021). The Lean startup framework: Closing the academic–practitioner divide. Entrepreneurship Theory and Practice.

[CR123] Shepherd DA, Patzelt H (2022). A call for research on the scaling of organizations and the scaling of social impact. Entrepreneurship Theory and Practice.

[CR124] Shepherd DA, Williams T (2020). Entrepreneurship responding to adversity: Equilibrating adverse events and disequilibrating persistent adversity. Organization Theory.

[CR125] Silva DS, Ghezzi A, de Aguiar RB, Cortimiglia MN, ten Caten CS (2020). Lean startup, agile methodologies and customer development for business model innovation: A systematic review and research agenda. International Journal of Entrepreneurial Behavior & Research.

[CR126] Silva DS, Ghezzi A, de Aguiar RB, Cortimiglia MN, ten Caten CS (2021). Lean startup for opportunity exploitation: Adoption constraints and strategies in technology new ventures. International Journal of Entrepreneurial Behavior & Research.

[CR127] Sjödin D, Parida V, Jovanovic M, Visnjic I (2020). Value creation and value capture alignment in business model innovation: A process view on outcome-based business models. Journal of Product Innovation Management.

[CR128] Snihur Y, Clarysse B (2022). Sowing the seeds of failure: Organizational identity dynamics in new venture pivoting. Journal of Business Venturing.

[CR129] Snihur Y, Wiklund J (2019). Searching for innovation: Product, process, and business model innovations and search behavior in established firms. Long Range Planning.

[CR130] Snyder H (2019). Literature review as a research methodology: An overview and guidelines. Journal of Business Research.

[CR131] Sosna M, Trevinyo-Rodríguez RN, Velamuri SR (2010). Business model innovation through trial-and-error learning: The Naturhouse case. Long Range Planning.

[CR132] Spieth P, Schneckenberg D, Ricart JE (2014). Business model innovation–state of the art and future challenges for the field. R&D Management.

[CR133] Spieth P, Schneider S, Clauß T, Eichenberg D (2019). Value drivers of social businesses: A business model perspective. Long Range Planning.

[CR134] Sull DN (2004). Disciplined entrepreneurship. MIT Sloan Management Review.

[CR135] Teece DJ (2010). Business models, business strategy and innovation. Long Range Planning.

[CR136] Teece DJ (2018). Business models and dynamic capabilities. Long Range Planning.

[CR137] Thomke S (2020). Building a culture of experimentation. Harvard Business Review.

[CR138] Thorgren S, Williams TA (2020). Staying alive during an unfolding crisis: How SMEs ward off impending disaster. Journal of Business Venturing Insights.

[CR139] Timmers P (1998). Business models for electronic markets. Electronic Markets.

[CR140] Troisi O, Maione G, Grimaldi M, Loia F (2020). Growth hacking: Insights on data-driven decision-making from three firms. Industrial Marketing Management.

[CR141] Vargo SL, Lusch RF (2004). Evolving to a new dominant logic for marketing. Journal of Marketing.

[CR142] Visnjic I, Wiengarten F, Neely A (2016). Only the brave: Product innovation, service business model innovation, and their impact on performance. Journal of Product Innovation Management.

[CR143] Wenzel, M., Stanske, S., & Lieberman, M. B. (2021). Strategic responses to crisis. *Strategic Management Journal, 42*(2).

[CR144] Whetten DA (1989). What constitutes a theoretical contribution?. Academy of Management Review.

[CR145] Williams TA, Gruber DA, Sutcliffe KM, Shepherd DA, Zhao EY (2017). Organizational response to adversity: Fusing crisis management and resilience research streams. Academy of Management Annals.

[CR146] Wirtz BW, Pistoia A, Ullrich S, Göttel V (2016). Business models: Origin, development and future research perspectives. Long Range Planning.

[CR147] Zellweger, T. M., & Zenger, T. R. (2021). Entrepreneurs as scientists: A pragmatist approach to producing value out of uncertainty. *Academy of Management Review*, in press. 10.5465/amr.2020.0503

[CR148] Zott C, Amit R, Massa L (2011). The business model: Recent developments and future research. Journal of Management.

[CR149] Zuzul T, Tripsas M (2020). Start-up inertia versus flexibility: The role of founder identity in a nascent industry. Administrative Science Quarterly.

